# Ball Milling’s Effect on Pine Milled Wood Lignin’s Structure and Molar Mass

**DOI:** 10.3390/molecules23092223

**Published:** 2018-09-01

**Authors:** Grigory Zinovyev, Ivan Sumerskii, Thomas Rosenau, Mikhail Balakshin, Antje Potthast

**Affiliations:** 1 Division of Chemistry of Renewable Resources, Department of Chemistry, University of Natural Resources and Life Sciences, Konrad-Lorenz-Strasse 24, A-3430 Tulln, Austria; grigory.zinovyev@boku.ac.at (G.Z.); ivan.sumerskii@boku.ac.at (I.S.); thomas.rosenau@boku.ac.at (T.R.); antje.potthast@boku.ac.at (A.P.); 2 Department of Bioproducts and Biosystems, School of Chemical Engineering, Aalto University, P.O. Box 16300, FI-00076 Aalto, Finland

**Keywords:** ball milling, carbohydrate composition, characterization, functional groups, milled wood lignin, molar mass distribution, softwood

## Abstract

The effect of ball milling expressed as the yield of milled wood lignin (MWL) on the structure and molar mass of crude milled wood lignin (MWLc) preparation is studied to better understand the process’ fundamentals and find optimal conditions for MWL isolation (i.e., to obtain the most representative sample with minimal degradation). Softwood (loblolly pine) MWLc preparations with yields of 20–75% have been isolated and characterized based on their molar mass distribution (by Size Exclusion Chromatography (SEC)), hydroxyl groups of different types (^31^P NMR), methoxyl groups (HS-ID GC-MS), and sugar composition (based on methanolysis). Classical MWL purification is not used to access the whole extracted lignin. The results indicate that lignin degradation during ball milling occurs predominantly in the high molar mass fraction and is less pronounced in the low molar mass fraction. This results in a significant decrease in the M_z_ and M_w_ of the extracted MWLc with an increase in the yield of MWLc, but has only a very subtle effect on the lignin structure if the yield of MWLc is kept below about 55%. Therefore, no tedious optimization of process variables is necessary to achieve the required MWLc yield in this range for structural studies of softwood MWL. The sugar composition shows higher amounts of pectin components in MWLs of low yields and higher amounts of glucan and mannan in high-yield MWLs, confirming that lignin extraction starts from the middle lamella in the earlier stages of MWL isolation, followed by lignin extraction from the secondary wall region.

## 1. Introduction

For a detailed characterization of native lignin polymers, its isolation from the accompanying matrix is still required. Although sophisticated methods that can work on solid or swollen states are available, most methods require analysis of the fully dissolved form. This, in turn, requires an isolation of soluble (lignin) preparations. Such isolation protocols for lignins are usually based on ball milling and date back to the mid-1950s when Björkman [1] developed a protocol based on the extraction of extensively ball milled wood by neutral solvents at room temperature (i.e., milled wood lignin (MWL)). Since then, the original procedure has been widely applied throughout the wood chemistry community and further modified to increase lignin yields while minimizing structural alterations [2,3,4]. The MWL usually serves as a standard lignin considered close to the native structure of lignin in the respective raw material used. To further reduce the isolation-induced modifications, Chang et al. suggested a procedure called cellulolytic enzyme lignin (CEL) [3]. CEL is obtained in higher yields, compared to MWL of the same milling duration, and therefore is less degraded. Hence, it is considered more representative of the total lignin fraction in wood. To further maximize the yield of isolated lignin, Guerra et al. studied a more complex procedure (suggested earlier by researchers [5]) called enzymatic mild acidolysis lignin (EMAL) that combines milling, enzymatic treatment, and an additional acidolysis step [4]. Eventually, Lu and Ralph [6], as well as Wang et al. [7], suggested procedures to completely dissolve the whole plantcell walls (CW) by applying ionic liquids with a high dissolution power, and thus allow for analysis via multidimensional nuclear magnetic resonance (NMR). Utilizing Lu and Ralph’s CW dissolution protocol, Sixta et al. [8] suggested some modified procedures to isolate lignin with a high degree of purity from dissolved CW. Sixta and co-workers precipitated acetylated lignin after dissolution in ionic liquids with an average yield of about 50%. Capanema et al. [2] regenerated whole wood from the ionic liquid and removed the more readily accessible carbohydrate fraction by enzymatic hydrolysis. The obtained regenerated CEL preparation (RCEL) showed a yield above 90% for the isolated lignin, based on the wood lignin, and little carbohydrate content (below 5%). 

Each of these protocols has benefits and disadvantages, and each is well suited to specific uses. For example, CW preparations allow access to all cell wall components to elucidate structural features by multidimensional NMR in the presence of degraded carbohydrates, including a semi-quantitative characterization of the side chain moieties, but do not allow quantifying the functional group composition or analyzing quaternary carbon moieties or molar mass information. 

However, classical MWL with a low amount of carbohydrate impurities, and hence good solubility, can be used in a wide array of wet chemistry and spectroscopic methods. The drawback is usually the relatively low yield and uncertainty of how representative the MWL is of the whole wood lignin in situ. When different lignin preparations were compared, no significant differences have been found between classical softwood MWL and CEL by quantitative ^13^C NMR [9,10], and neither between MWL, CEL, and CW [11,12]. Furthermore, MWL, CEL, and EMAL are rather similar when analyzed by ^31^P NMR and derivatization followed by reductive cleavage (DFRC)-^31^P NMR [4]. Chang et al. reported CEL to be structurally similar to MWL, but to have a significantly higher molar mass and a lower phenolic group content [3]. Balakshin et al. found that the β-*O*-4 content (indicative of the degree of degradation) increases from the first fraction removed in the first purification step for crude MWL (MWLc) to the purified stage, and finally to the CEL fraction (22.2, 27.5, 30.5, 35.1/Ar in AcOH-LCC, MWLc, MWLp, CEL, respectively) [13]. Larger variances between MWL and CEL were observed for hardwood lignin (maple) due to *per se* higher variations in the lignin structure of the hardwood cell wall’s different morphological regions [2].

To attempt a translation of the structure of an isolated lignin preparation into that of the original native lignin *in situ*, we must consider two questions: how significant are the changes during isolation, and how well does the isolated lignin fraction represent the total wood lignin? Hence, it should be noted that lignin preparations’ degree of representability does not necessarily correlate with the (isolated) lignin yield. For example, CEL with a 25% yield has been found to be more representative than an MWL with a 50% yield [2]. Apparently, CEL preparation encompasses the lignin from different regions of the whole cell wall (in correct proportions), while the MWL might be more representative of a specific region of the cell wall, such as the middle lamella.

Since all lignin preparations, including those used for direct dissolution in ionic liquids, require extensive ball milling, the question of how this mechanical treatment affects the lignin structure is a critical one. It has been postulated that the degree of lignin degradation does not depend on aerobic or anaerobic conditions, but solely on the duration and vigor of milling [14]. From these results, it can be concluded that primary tribochemical processes dominate over secondary radical processes that involve atmospheric oxygen and subsequent radical reactions. Furthermore, Fujimoto et al. [14] introduced the yield of ‘extractable lignin’ determined by a 96% dioxane extraction of the milled wood, followed by UV analysis; this should be similar to the gravimetric yield of MWLc corrected for carbohydrate content. It was suggested that the yield is, therefore, a better indicator of the extent/vigor of ball milling than the milling duration alone [14].

Although the whole wood lignin significantly degrades during milling, which is detected by strongly diminished β-*O*-4 numbers [10,14], the situation for isolated MWL is very different. Balakshin and Capanema did not find significant differences in lignin structure for traditional (30%) and high yields (70%) of purified MWLs based on ^13^C NMR of acetylated MWL [15,16]. Similarly, Hu et al. did not find any significant dissimilarities between purified MWL with about a 30% yield and CEL isolated with yields of 20–86% analyzed by quantitative ^13^C NMR [10].

There are only a few systematic studies of the lignin structure versus the isolation yield or milling duration while other variables are kept constant. For softwood, Hu et al. have not found any differences in the β-*O*-4 content of MWLc in the yield range of 15–46%. In a comprehensive study of EMAL (by ^31^P NMR and DFRC-^31^P NMR), Guerra et al. reported dramatic lignin degradation when ‘intensive milling’ in a vibratory mill for several days is used and no degradation when ‘mild milling’ in a slow rotary mill for up to 34 days is applied [4]. Because the conditions for enzymatic hydrolysis and acidolysis are the same, the structural changes in EMAL are solely associated with the differences in ball milling. Related to hardwoods, the severe degradation of β-*O*-4 linkages, analyzed by ozonolysis, in MWLc in the yield range of 7–52% were reported by Fujimoto [17], which is in stark contrast to the results by Capanema et al. [2]. In the latter paper [2], no β-*O*-4 degradation was observed with a combination of quantitative ^13^C and 2D-NMR of MWLc from maple. These examples show that there are significant discrepancies among published studies related to MWLc structural changes during ball milling, and that evidently additional work is needed to further clarify the situation.

The molar mass of native lignin is likely a much more sensitive indicator of chemical changes than the chemical structure or linkage ratios. Imagine that forming one single bond between two similar lignin molecules would be almost impossible to detect by linkage analysis or NMR scrutiny, but would double the molecular weight. Molecular weight analysis, provided it is reliable and highly reproducible, would thus be able to report changes in the native lignin structure much more trustworthily than the alternative lignin analysis techniques. Both the degradation of the lignin by tribochemical fragmentation and polymerization via the recombination of generated radicals are *per se* reasonable assumptions, especially when extensive milling is applied, and molecular weight analysis would seem to be a method of choice to at least better understand the situation. To be fully conclusive, a molar mass analysis must be performed under the premises of molecularly disperse solution and a separation free from secondary effects, such as aggregation and column interaction. These conditions are not always easy to ensure and prove, regardless of which setup is used. However, a reliable setup should at least be able to provide a comparison of samples measured within the same system. Unfortunately, there is little data available on changes in the molecular weight distribution (MWD) of MWL during milling. Guerra et al. reported an increase in the molar mass of EMAL with increasing milling duration; however, the results were likely affected by aggregation effects [4]. 

The analysis of MWLc should clarify the issues discussed above and provide a better understanding of the structural changes caused by the MWL isolation protocol. This will be useful in understanding the ball milling’s effect on the molar mass and structure of preparations, and should provide an optimized procedure that can obtain the most representative lignin with minimal structural modifications. In this account, we would like to communicate our attempts to use molar mass analysis, along with ^31^P NMR, HS-ID GC-MS, and carbohydrate composition analysis of the MWLcs, to address changes in lignin structure upon MWL preparation. 

## 2. Results and Discussion 

Currently, MWL is the most common preparation used to mimic a native lignin structure that can be used to study the reactivity of native lignin *ex planta*. It is well recognized that MWL represents only a portion of native lignin in a wood cell wall. It has also been demonstrated that lignin could undergo structural changes during the MWL isolation process, especially during milling. However, few quantitative relationships have been found between structural changes in lignin and the degree of milling [2,10].

### 2.1. Effect of Milling Conditions on the Yield of Milled Wood Lignins

**Figure 1:**
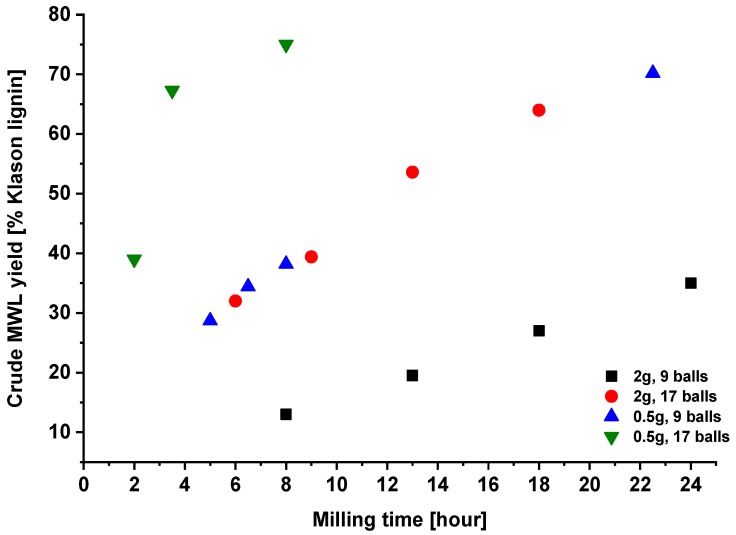
The effect of the number of balls and amount of wood material used per milling container.

Milling duration is not the only variable affecting the yield of MWLc. For the same milling equipment used, the sawdust load in the milling jar and the number of balls used had strong effects on the yield (Figure 1). As only selected samples were analyzed for the carbohydrate content and the data was not available for all MWLc, the yield of MWLc was not corrected for the carbohydrate amounts in the sample. Milling time plotted included only true milling, without pause between milling cycles. As the number of balls increased and the amount of material decreased, a certain yield was reached within a shorter period (Figure 1). More balls and less wood content assure shorter milling durations to reach the target yield. Inert ball and container materials, such as zirconium oxide, should be preferred to avoid contamination with metal ions. The milling cycles applied and the ball material also affect the yield, as well as the ball size and humidity of the material. In the present study, we selected the following MWLc samples: P-20 (2 g sawdust, 9 balls, 13 h milling), P-38 (0.5 g sawdust, 9 balls, 8 h milling), P-32, P-39, and P-54 (2 g sawdust, 17 balls; 6, 9 and 13 h milling, respectively), P-67, and P-75 (0.5 g sawdust, 17 balls, 3.5 and 8 h milling, respectively). Therefore, we followed the work by Fujimoto et al. [14] to reflect milling severity, and hence, have related all our data to the corresponding yield of the isolated MWLc preparation. 

### 2.2. Molar Mass Distribution

**Figure 2:**
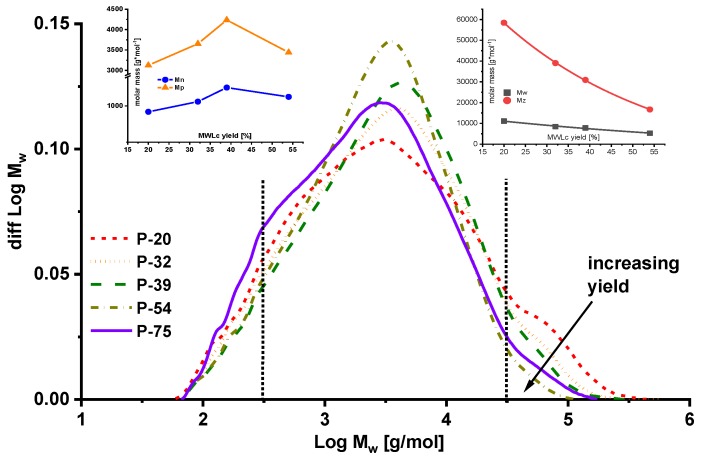
Molar mass distribution in relation to the crude lignin yield. Inserts: Corresponding statistical moments M_n_ and M_p_ (**left**) and M_z_ and M_w_ (**right**).

**Figure 3:**
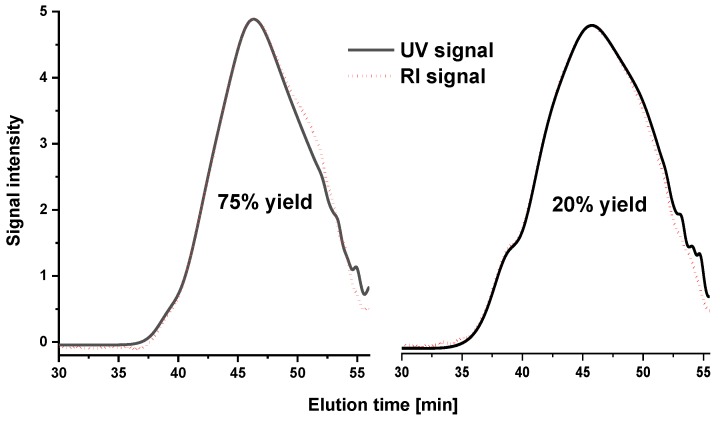
Comparison of chromatograms (UV vs. RI traces) for a 75% crude lignin yield (**left**) and a 20% crude lignin yield (**right**).

Figure 2 provides the molecular weight distribution of lignins in a crude yield ranging from 20% to 75%. The high molar mass shoulder clearly decreased with the increasing yield, the molar mass following an exponential decrease of the weight average and the z-average molar mass moments (insets in Figure 2). The latter was especially sensitive to changes occurring in the high molar mass range. Distinguishing between aggregates and real high molar mass structures is not trivial unless light scattering data is available. Still, in the present case, we are positive about the effect being indeed correlated to high molar mass lignin and reflecting the milling effect on molar mass at a clear trend towards lower MWL yields. Figure 3 presents an overlay of the two concentration-dependent signals for refractive index (RI) and UV. The UV signal reported lignin molecules, while the RI signal accounted for the sum of lignin and carbohydrates/pectin moieties. Both signals ran congruently in the high molar mass range, from which we can conclude that the structures destroyed with increasing severity mainly originate from lignin rather than including carbohydrates, even considering the overall lower concentration of carbohydrates and the lower sensitivity of the RI signal. Small differences in the two signals occurred in the low molar mass range below 500 Da. 

### 2.3. Functional Groups and Sugar Composition

**Figure 4:**
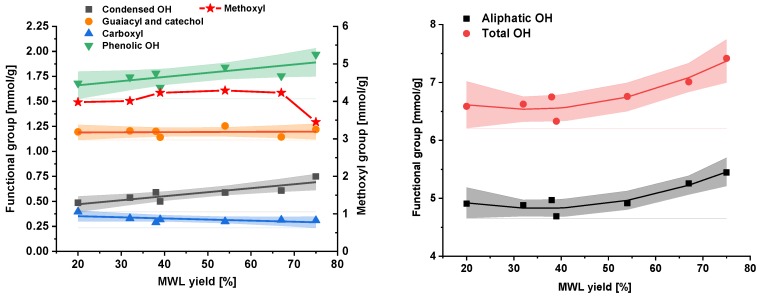
Functional groups of crude MWL depending on the milling yield. Shadowed areas indicate the 95% confidence interval.

Figure 4 displays the functional groups depending on crude lignin yield. One can see that MWL samples produced under different milling conditions (Figure 1) fit the same correlation curves when plotted versus the yield of MWLc, confirming once again the original finding [14]. Moreover, samples P-38 and P-39 obtained under different milling conditions (number of balls and milling time), but with practically the same yield (Figure 1), had very similar functional group composition (Figure 4). 

**Figure 5:**
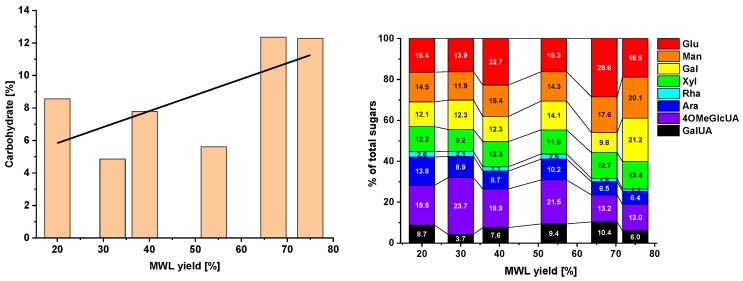
Carbohydrate contents (**left**) and distributions of individual sugars (**right**) depending on the crude MWL yield.

Changes in functional groups in the MWLc yield range of 20–75% were very minor. The methoxyl (OMe) groups (Figure 4, left) were probably not affected by the milling *per se*, as they behave like small side chains, which are usually not cleaved in topochemical processes [14]. With the amount of methoxyls in softwood hemicelluloses being rather small, the OMe groups primarily reported the lignin content. The hydroxyl groups, however, were sum parameters for aliphatic OH in lignin (Figure 4, right) and carbohydrates, which increased beyond a 55% lignin yield by about 11% (Figure 5, left). The phenolic OH groups were affected by cleavage reactions within the lignin molecule, which correspond to molar mass data. The amount of phenolic OH increased by about 15%; guaiacyl and catechol units remained constant, while the amount of condensed phenolic hydroxyls changed by about 25% in the yield range of 20–67%, plus another 25% in the yield range of 67–75% (Figure 4, left).

The carbohydrate content and composition in each yield fraction are presented below (Figure 5). The composition’s dependence on the yield of MWLc was rather complex; however, some general tendencies were observed. The amount of pectin components, such as uronic acids (galacturonic acid, GalUA, and 4-*O*-methyl-glucuronic acid, 4OMeGlcUA) and arabinan, decreased with increasing MWLc yield, while the sum of mannan, galactan, and glucan increased; the amount of xylan was rather constant. This correlates well with earlier studies when LCC-rich fractions (called LCC-AcOH) were isolated by fractionation of similar pine MWLc of normal and high yields [13]: the LCC isolated from the high-yield MWLc contained lower amounts of pectin components and higher amounts of main polysaccharides. The same tendency was observed with the whole (non-fractionated) MWLc (Figure 5). Thus, our data confirmed that lignin extraction starts from the middle lamella in the earlier stages of MWL isolation, followed by lignin removal from the secondary wall region. It is noteworthy that the changes in the carbohydrate content and composition were much more pronounced above MWLc yields of ca. 55% (Figure 5). P-67 and P-75 samples contained significantly higher amounts of carbohydrates and their share of pectin components was significantly lower than in samples P-20–P-54. 

Thus, our finding that lignin degradation occurred predominantly in high molar mass lignin fractions and rather leveled off for low molar mass lignin fractions explains well that significant degradation of the total wood lignin was observed during milling [10,14], but was very limited for the MWLc. All lignin degradation very likely occurred before the MWL was extracted (in higher molar mass lignin fractions insoluble yet in 96% dioxane), and therefore, was not readily visible in the structure of MWL (i.e., lower molecular mass fractions of the total lignin).

Our results on the analysis of main lignin functionalities with ^31^P NMR correlate well with earlier studies on the effect of milling on the structure of lignins analyzed with quantitative ^13^C and 2D NMR [2,10,13,15,16]. Therefore, we did not repeat detailed NMR analysis (quantitative 2D NMR) in this study. However, it can be useful if some minor lignin structures are focused on. In particular, the effect of milling severity on the structure of the whole CW lignin analyzed by 2D NMR [11,12] is of interest for further studies.

Our results on lignin degradation differ significantly from EMAL studies employing similar analyses (^31^P NMR). The structure of pine EMAL showed severe lignin degradation after 100 h of milling, which was required to reach an EMAL yield of 75% [4]. The amount of phenolic OH and carboxyl groups in EMAL almost doubled, whereas we observed only a 15% increase in phenolic OH and even a 25% decrease in carboxyl groups. Understandably, Guerra et al. explained these changes as being caused by the intensive milling [4]. However, our results clearly show this is not the case as we reached this yield in a much shorter duration (i.e., with a significantly higher intensity). As has been suggested earlier, the degradation in the EMAL protocol may not be connected to ball milling, but rather to the subsequent acidolysis step [15]. The actual mechanism of degradation remains hypothetical, but the milled lignin is more prone to acid-induced degradation reactions in the EMAL protocol. Mild extraction with neutral dioxane at room temperature in the MWL protocol avoids such processes to large extent. These observations result also in a rather practical outcome; because ‘intensive milling’ does not significantly affect the structure of MWLs, the process can be intensified and, hence, accelerated. A representative MWL preparation can be obtained within a few hours of milling. Applying an accelerated solvent extraction of the milled wood allows the generation of an MWL preparation within one working day [15].

## 3. Conclusions

Lignin degradation during softwood ball milling occurred predominantly in the high molar mass fraction and was less pronounced in the low molar mass fraction. This resulted in a significant decrease in the M_z_ and M_w_ of the extracted MWLc with an increase in the yield of MWLc, but had a considerably smaller effect on lignin structural characteristics when the yield of MWLc was kept below about 55%. Although lignin extraction started from the middle lamella in the earlier stages of MWL isolation, followed by lignin extraction from the secondary wall region, it did not have a significant effect on the main structural characteristics of softwood MWL if the yield ranged between 20% and 55%. Therefore, no tedious optimization of process variables was necessary to achieve the required MWLc yield in this range. Furthermore, as intensive ball milling did not have strongly negative effects on the structure of MWLs, in contrast to earlier studies of the EMAL isolation method, the MWL isolation protocol could be greatly accelerated to produce an MWL preparation within one working day.

## 4. Materials and Methods 

### 4.1. Isolation of Milled Wood Lignin Preparations 

Loblolly pine sawdust (fraction < 60 mesh) was extracted with ethanol/benzene (*v*/*v*, 1:2) prior to the isolation of MWL preparations. 

### 4.2. Crude Milled Wood Lignin

Crude MWLs were isolated according to the classical method [18] modified recently [14]. A planetary ball mill (Pulverisette 7, Fritsch, Idar-Oberstein, Germany) was used, in contrast to a vibratory ball mill in Bjorkman’s protocol. The extracted sawdust (0.5 or 2 g) was subjected to milling (at various times) at 600 rpm using ZrO_2_ bowls and 9 or 17 ZrO_2_ balls. Milling was performed at 600 rpm, pausing 10 min after each 30 min period of milling to avoid heating above 40 °C (controlled with an IR thermometer). The wood meal obtained was extracted with dioxane (96% *v*/*v*), and the solvent was then evaporated under vacuum at 35 °C. To remove traces of dioxane, a few drops of H_2_O were added to the solid matter and evaporated again. This procedure was repeated three times. Finally, the solid matter was dried in a vacuum oven at 35 °C to obtain MWLc preparations.

### 4.3. Sugar Analysis 

Carbohydrate analysis was performed according to Sundberg et al. [19]. MWLc samples (ca. 20 mg) underwent methanolysis in 2 mL of 2 M methanolic HCl by heating to 100 °C for 5 h. Sorbitol (100 µL, 5 mg mL^−1^ in methanol) was added as an internal standard (IS). The samples were silylated and analyzed by GC. Carbohydrate contents were presented as polysaccharides (anhydrosugars). 

### 4.4. Molar Mass Analysis

Non-derivatized lignins dissolved in DMSO/LiBr were used for molar mass distribution analysis to avoid any undesirable effect from lignin derivatization discussed earlier [20].

Size-exclusion chromatography (SEC) was performed on a Dionex UltiMate 3000 (Thermo Fisher Scientific, Dreieich, Germany) with an autosampler, a column oven, a UV detector and a coupled Shodex RI-101 RI detector. The separations were carried out through an Agilent PLgel guard column (7.5 × 50 mm) and three Agilent PolarGel M columns (7.5 × 300 mm) in a series. Dimethylsulfoxide (DMSO) with 0.5% (*w*/*w*) lithium bromide (LiBr) was used as the eluent. Column calibration was performed with a set of narrow poly(styrene sulfonate) sodium salt (Na-PSS) standards of known molecular weights (M_w_ range, 1100–148,000 g/mol; PD < 1.20). To achieve a complete dissolution of Na-PSS standards in DMSO, the samples were pre-treated with cation exchange resin according to the literature [20]. DMSO was HPLC grade and supplied by Sigma-Aldrich Chemie GmbH (Schnelldorf, Germany). Na-PSS standards were supplied by PSS Polymer Standards Service GmbH (Mainz, Germany). The analysis parameters were as follows: flow rate: 0.5 mL/min, column temperature: 40 °C, injection volume: 10 μL, UV detector at 280 nm, and RI detector at 35 °C. Data evaluation was performed with Chromeleon software, version 6.80. The lignin samples were dissolved in an isolated form (without derivatization) at r.t. in the SEC eluent (10 mg/mL), shacked overnight, and filtered through a 0.45 μm PTFE syringe filter before injection. 

### 4.5. Functional Groups Analysis 

The amounts of different OH and COOH groups were determined by ^31^P NMR spectroscopy according to Granata and Argyropoulus [21] with the modification by Korntner et al. [22]. About 25 mg of MWLc was completely dissolved in 700 μL of a 1:1.6 mixture of CDCl_3_ and pyridine (nondeuterated). For all samples, dissolution was achieved by shaking only; no ultrasonic bath, heating, or other dissolution auxiliaries were applied. Furthermore, 200 μL of a stock solution, containing IS, *N*-hydroxy-5-norbornene-2,3-dicarboxylic acid imide (e-HNDI), (0.02 mmol mL^−1^) and relaxation agent, chromium acetylacetonate [Cr(acac)_3_; 5 mg ml^−1^], were added. After thorough mixing, 100 μL of phosphitylation reagent [2-chloro-4,4,5,5-tetramethyl-1,3,2-dioxaphospholane (TMDP)] was injected through a septum into the vial to avoid contact of the reagent with moisture. The sample was shaken for 1 h at r.t. and then transferred into an NMR tube. The spectra were acquired using a Bruker Avance II 400 (^1^H resonance at 400.13 MHz, ^13^C at 100.61 MHz and ^31^P at 162 MHz) equipped with a 5 mm broadband observe probe head (BBFO); z-gradient at r.t. (standard Bruker pulse programs). A 0.6 s acquisition time and a relaxation delay of 15 s were used and 256 scans were collected. ^31^P data were collected with 64 k data points and apodized with an exponential window function (lb = 5) before Fourier transformation. Integration was done after automatic baseline correction (Bruker TopSpin) according to Korntner et al. [22].

Methoxyl group analysis was performed according to Sumerskii et al. [23]. Approximately 1 mL of HI acid was added to a 10 mL headspace screw cap vial, containing the MWL sample (5–10 mg), internal standards 4-methoxybenzoic acid-d_3_ (4–5 mg) and 4-ethoxybenzoic acid-d_5_ (2–3 mg), and a small magnetic stirring bar. The vials were tightly closed with screw caps equipped with PTFE-covered silicon septa and heated for 3 h at 110 °C. After cooling, 4 mL of H_2_O were injected through the septa. GC-MS analysis was carried out on an Agilent 6890 N gas chromatograph coupled to an Agilent 5975B inert XL mass selective detector (MSD). The GC was equipped with a split-/splitless inlet and DB5-ms column (i.d. (30 m × 0.25 mm) × film thickness (0.25 µm); J&W Scientific, Folsom, CA, USA). The split-/splitless inlet operated under the following conditions: constant column flow: 1.0 mL min^−1^ using He carrier gas, split-ratio: 1:50, injector: 250 °C. Oven temperature gradient profile: 40 °C (2 min), 10 °C min^−1^ to 150 °C (3 min) and back to initial values. The MSD was operated in the Electron Ionization (EI) mode at 70 eV ionization energy and 1.13 × 10^−7^ Pa. Ion source temperature: 230 °C, quadrupole: 150 °C, transfer line: 280 °C. The data were acquired in the selected ion monitoring (SIM) mode at 50 ms dwell time for each ion group. A closed loop headspace sampler (Agilent Technologies 7697A equipped with a 20 µL loop) was used for injections. The sampler operated under the following conditions: vial temperature: 50 °C, loop temperature: 60 °C, transfer line temperature: 70 °C, vial equilibration time: 3 min, vial pressurization time: 0.2 min, loop fill time: 0.18 min, loop equilibration time: 0.05 min, injection time: 1 min. 

The error bars in the figures, if not mentioned otherwise, are SDs from the mean values (duplicate measurements).
